# Seismic Assessment and Retrofitting of Existing Road Bridges: State of the Art Review

**DOI:** 10.3390/ma15072523

**Published:** 2022-03-30

**Authors:** Dominik Skokandić, Anđelko Vlašić, Marija Kušter Marić, Mladen Srbić, Ana Mandić Ivanković

**Affiliations:** Department for Structures, Faculty of Civil Engineering, University of Zagreb, 10 000 Zagreb, Croatia; andjelko.vlasic@grad.unizg.hr (A.V.); marija.kuster.maric@grad.unizg.hr (M.K.M.); mladen.srbic@grad.unizg.hr (M.S.); ana.mandic.ivankovic@grad.unizg.hr (A.M.I.)

**Keywords:** seismic assessment, retrofitting, existing road bridges, bridge assessment, Croatia earthquake, urgent strengthening

## Abstract

The load-carrying capacity assessment of existing road bridges, is a growing challenge for civil engineers worldwide due to the age and condition of these critical parts of the infrastructure network. The critical loading event for road bridges is the live load; however, in earthquake-prone areas bridges generally require an additional seismic evaluation and often retrofitting in order to meet more stringent design codes. This paper provides a review of state-of-the-art methods for the seismic assessment and retrofitting of existing road bridges which are not covered by current design codes (Eurocode). The implementation of these methods is presented through two case studies in Croatia. The first case study is an example of how seismic assessment and retrofitting proposals should be conducted during a regular inspection. On the other hand, the second case study bridge is an example of an urgent assessment and temporary retrofit after a catastrophic earthquake. Both bridges were built in the 1960s and are located on state highways; the first one is a reinforced concrete bridge constructed monolithically on V-shaped piers, while the second is an older composite girder bridge located in Sisak-Moslavina County. The bridge was severely damaged during recent earthquakes in the county, requiring urgent assessment and subsequent strengthening of the substructure to prevent its collapse.

## 1. Introduction

The civil infrastructure network in the United States and Western Europe was built primarily in the post-World War II era, most notably in the 1960s and 1970s, and has therefore reached the end its designed service life. In addition to the ageing process, the deterioration of materials along with increased traffic volume and weight contribute to the alarming condition of critical infrastructure. Due to their complexity and exposure, bridges and viaducts are often defined as critical parts of transport networks, and many were built according to old codes that did not impose such strict durability requirements as today [1]. Although the evaluation and maintenance of bridges has been the subject of numerous research projects and papers over the past two decades, public awareness of the safety of existing bridges has been heightened by the two recent catastrophic collapses. The first was the Morandi Bridge in Genoa [2], which collapsed during a storm, and the second was a pedestrian overpass in Miami [3] that collapsed during construction, both in 2018. In both cases, the collapse resulted in multiple fatalities and enormous property damage. These types of bridge failures do not occur often; however, because of the serious consequences they attract widespread attention and are often used as lessons for the future. One of the causes which can trigger the partial or complete collapse of deteriorated existing bridges is an extreme natural event, such as flooding, landslides, or earthquakes, as presented in [4].

Bridges in seismically active areas are sensitive to the effects of earthquakes, most notably displacements and vibrations due to ground acceleration. Therefore, they must be appropriately designed and constructed with a certain degree of ductility.

The current design standard for new bridges in the EU, the Eurocodes [5], provides an approach based on modal analysis and behavioral factors. This means that it is uneconomical to design bridges that provide a full elastic response to seismic actions. As a solution, the design method takes into account the reduced seismic loads (with a behavioral factor) while focusing on structural detailing to ensure ductile behavior of the bridge system [6]. While such an approach is justified for the design of new bridges, it is not cost-effective when used in the assessment of existing ones which were designed according to old codes. These codes did not include guidelines for ductile behavior or structural robustness, and older codes did not consider seismic actions at all. As the Eurocode does not cover the assessment of existing bridges, the purpose of this paper is to provide the state of the art with respect to the methods used by researchers and engineers worldwide.

In many cases, design codes and strategies for bridge maintenance have been modified and improved after a major earthquake. In California, for example, the Department of Transportation initiated a bridge retrofit program after the 1971 San Fernando earthquake, and after the 1989 Loma Prieta earthquake this program was made mandatory for all public bridges in the state of California [7]. In Japan, the catastrophic Great Hanshin Earthquake in 1995 resulted in the complete collapse of the 18-span highway bridge in Kobe. Due to the extreme cases of structural failure during the Kobe earthquake, the lessons learned have influenced design codes both in Japan and worldwide [8].

In Croatia, most of the medium and large span road bridges were built between the 1960s and 1980s using the design codes that took into account a proportion of the seismic load [9]. Therefore, they need to be re-evaluated and retrofitted due to their age, deterioration, the aggressive marine environment, increased traffic load, improper maintenance, etc. On the other hand, there are a number of short-span bridges on some local and less frequented state roads that were built before seismic loads were part of the design specifications. These bridges are in many cases supported by masonry abutments and/or piers, while the superstructure consists of either steel girders or reinforced concrete slabs. Several similar bridges are located in Sisak-Moslavina County (SMC), one of the largest counties in Croatia, located about 50 km southeast of Zagreb. Both the city of Zagreb and SMC were hit by strong earthquakes in 2020. In March 2020, a strong earthquake hit Zagreb and its surroundings, followed by numerous aftershocks. The epicenter was located about 7 km from the city center, the magnitude was M_L_ = 5.5, and the intensity according to the EMS−98 scale was VII [10]. The second earthquake occurred on 29 December, with an epicenter about 3 km from the town of Petrinja in Sisak-Moslavina County. The magnitude of the earthquake was M_L_ = 6.2, and it had an intensity between VIII and IX according to the EMS−98 scale [11]. Both earthquakes resulted in massive property damage and, unfortunately, fatalities. The total financial damage as estimated by the World Bank was around EUR 16.5 billion [12].

This paper is conceived as a state-of-the-art literature review focused on the methods for seismic assessment, analysis, and retrofitting of existing road bridges. Additionally, two Case Study bridges are presented as examples of the application of the described methods. Both bridges are located in Croatia, and they represent different approaches to seismic assessment, namely, as a part of regular maintenance in the first case and urgent repair following a significant earthquake in the second.

The first bridge is a reinforced concrete hinged strut frame bridge over four spans [13] located in the coastal part of Croatia. The second bridge is a two-span composite girder bridge located in Sisak Moslavina County, and was damaged during the 2020 earthquakes. Both bridges were built according to the old codes without taking into consideration the active seismic zones where they are located. The second bridge is one of eight bridges that were inspected after the December 2020 earthquake [14].

## 2. Seismic Assessment of Existing Road Bridges

### 2.1. Analysis Methods

The current EU seismic design standards, EN 1998, consist of six parts, the first of which addresses new buildings [15], the second of which addresses the seismic design of new bridges [5], and the third of which covers procedures and guidelines for the assessment and retrofit of existing buildings [16]. Parts four through six deal with special engineering structures such as silos, pipelines, foundations, towers, etc. The assessment of existing bridges is generally not covered by the current generation of Eurocodes, and EU Member States (and other countries around the world) use different approaches, as presented in [17]. During the assessment procedure for existing bridges, the greatest uncertainties are associated with the modelling of traffic loads, as these represent a dominant live load on the bridge structure [9,18]. However, seismic actions have a significant effect on bridges in seismic active areas, and can cause a sudden partial or complete failure if a capacity design methodology is not taken into account in the initial design [19].

Seismic evaluation is generally performed in a manner similar to the design of new bridges. It uses the numerical model of the existing bridge and site-specific seismic and soil data to define both the demand and the capacity of the bridge. This requires the use of one of the available seismic analysis methods listed in Table 1. Regardless of the choice of analysis method, the majority of existing bridges designed without consideration of seismic loads or with moderate seismic loads can be described as structures with inadequate lateral stiffness and ductility in their substructure elements, i.e., the piers and abutments.

In order to develop a numerical model of a bridge that does not deviate from realistic behavior, the first step in the assessment method is data collection. This includes the original design projects (if available in the archives), any previous general and special bridge inspection reports, and any records of previous strengthening or other work performed on the bridge [20]. For existing bridges, site-specific traffic load models can be extrapolated using Bridge Weigh-in-Motion methods along with additional structural information such as influence lines, dynamic properties, etc. [1]. Traffic measurements can provide modal properties (modal shapes, frequencies, etc.) of the bridge, as described in [21]. In addition to traffic and structural data, there are numerous other nondestructive (NDT) methods of collecting information on the properties of the built-in materials, the location and quantity of reinforcement, and numerous other bridge features [22]. These and similar methods are well-covered by a number of authors (e.g., [23,24,25,26]) and will not be described in detail, as they exceed the scope of this paper.

**Table 1:** List of the most commonly used methods for seismic analysis of existing road bridges.

Analysis Method	Type	Source
Response spectrum method	Linear analysis	[5,13,27]
Fundamental mode method		
Time series analysis		
Time history analysis	Non–linear analysis	[5,28]
Pushover analysis		[5,6,13,27,29,30,31]
Probabilistic and sampling methods	Non–linear analysis	[32,33,34,35]

The selection of an analysis method is based on a number of parameters, mainly the bridge type, as different structural systems are subjected to different failure modes. For example, masonry arch bridges and integral bridges are usually short-spanned, very massive, and have relatively squat piers. On the other hand, cable-stayed bridges, tall and long viaducts, and slender concrete arch bridges are much more sensitive to lateral forces and dynamic effects. In addition to the method of analysis, the performance of similar bridges under seismic loading depends on the number of piers, cross-section, type and amount of reinforcement, etc.

The disadvantage of linear modal analysis is that it does not consider the redistribution of forces that occurs after the development of the plastic joint(s). Therefore, it does not take into account the new corresponding failure modes, and consequently risks not identifying all the critical structural elements of the selected bridge [30]. On the other hand, a nonlinear analysis takes into account the formation of plastic joints and the corresponding seismic force which they dissipate through deformations. Such analyses are based on the rotational capacity of the structural elements, the M–φ curve, which exploits the nonlinear behavior of the material, leading to “hidden” reserves in the load-bearing capacity of the structural element and the corresponding bridge. On the other hand, this requires a high degree of knowledge about the behavior of the elements in terms of their rotational capacity [36]. For existing bridges, it is not always possible to provide sufficient information about the material properties and built-in reinforcement to develop an accurate M–φ curve. While NDT tools can be used to determine the material properties of the concrete [22], the amount and yield stress of the built-in reinforcement is often estimated from available documentation. The most common nonlinear analysis used in the assessment procedures for existing bridges is the static nonlinear analysis (pushover) method, as the dynamic method (cyclic loading-time history analysis) is time-consuming and requires data sets from realistic earthquakes.

Pushover analysis is performed by subjecting the numerical bridge model to a gradually increasing lateral force until the displacement reaches a predetermined threshold. It is convenient for existing structures where the predominant failure mode has been identified, because it does not consider higher modal shapes. Therefore, it can be used for most bridges for which the M–φ curve of structural elements is available or can be approximated.

Considering the large number of bridges in transportation networks, a number of authors have developed fragility curves for bridge stocks, which provide valuable data for bridge management and decision making in terms of prioritizing repairs and planning [37].

Fragility curves have proven to be a very convenient tool for evaluating typical bridges that make up the majority of the existing bridge stock in the EU and the US. For more complex bridge types, such as concrete arch bridges, cable-stayed bridges, and extremely long-span bridges in general, seismic evaluation again requires a more detailed analysis, as is always the case for landmark bridges. The most common bridge types on roads and motorways in the EU are reinforced (RC) or prestressed (PSC) concrete girder bridges with spans of up to 30 m, either simply supported or continuous over several spans, according to a survey conducted as part of the SERON research project [38]. Choi et al. [39] present an inventory of bridges in Central and Middle America with similar data. The most common superstructure material is reinforced concrete, followed by steel girders with concrete decking and PSC girders.

The seismic behavior of these bridges generally depends on the fragility of bearings, piers, abutments, and foundations, which should be analyzed using a nonlinear approach. The superstructure can generally be modeled with a linear elastic analysis, as its stiffness does not have a significant effect on the behavior of the bridge. In this analysis, it is assumed that the superstructure remains in the linear elastic region of the stress–strain curve during longitudinal seismic actions and transmits only the shear force to the substructure [39]. On the other hand, the piers and abutments should provide a significant measure of ductility and transversal stiffness in order to withstand seismic actions. Current design codes for new bridges [5] require high ductility levels for RC piers, achieved by confining the reinforcement. The ductile behavior in the compression zone of the cross-section should be ensured within the potential plastic hinge regions; at the same, time buckling of longitudinal reinforcement must be avoided, even after multiple cycles of seismic effects.

For existing bridges, which do not meet the listed detailing criteria, the seismic resilience (fragility curves) of RC piers should be developed based on the moment–curvature relationship, the M–φ curve. The M–φ curve for each cross-section can be developed analytically or numerically, if sufficient data is available, and subsequently used as an input in the nonlinear analysis. The procedure for the development of M–φ curves for existing RC piers with insufficient ductility is described in [6,36]. M–φ curves can be developed empirically using various scale models in laboratory experiments, as presented in [6,40].

### 2.2. Fragility Curves

Seismic fragility curves in general represent the ability of a structural system (e.g., a bridge) to withstand a seismic event. They provide a relationship between the capacity of the system (C) and demand (D), depending on the peak ground acceleration (PGA), in terms of the conditional probability of failure. Failure is defined as an event where the demand (D) exceeds the structural capacity (C) [41]. The fragility of the bridge can be expressed as

Fragility = P[D > C│PGA].(1)

**Figure 1:**
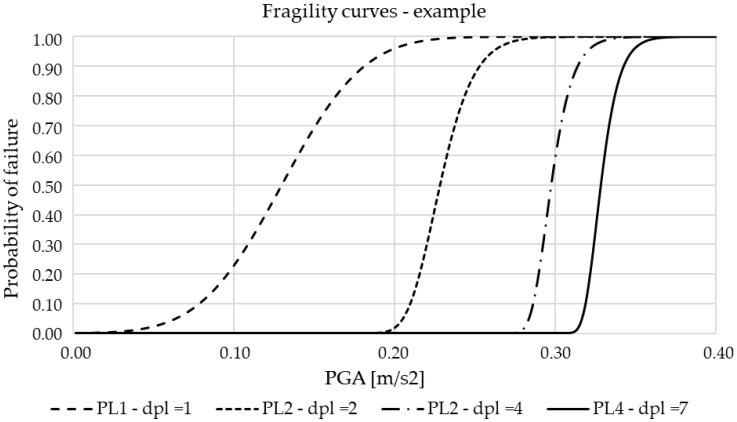
Example of fragility curves for bridge piers without adequate seismic capacity.

Fragility curves can be developed empirically based on historical data on past earthquakes or analytically using one of the methods listed in Table 1. The latter approach is more common, because there is insufficient data to empirically produce reliable curves [37]. Fragility curves are, in general, developed for one of the bridge components, mainly for the substructure parts, e.g., piers, abutments, and bearings. For each element, demand and capacity parameters are defined as a part of the analytical process in terms of displacements, lateral forces, rotation, etc., along with defined thresholds. A number of authors have dealt with this issue in the last two decades, as presented in detail in [37]. For example, Choi et al. [39] consider four limit states for bridge piers in terms of the damage index (d_pl_), which is defined as a ratio between the current horizontal displacement (e.g., of the top of the pier) and a displacement at the same point in a significant cross-section (e.g., the bottom of the pier). Theoretical fragility curves based on [39] are presented in Figure 1, each for one of the defined damage states.

### 2.3. Literature Review: Seismic Assessment

A number of authors have studied the seismic behavior of bridges as part of infrastructure networks, several of them using fragility curves. Choi et al. [39] analyzed a large bridge inventory in the U.S.; based on this, they defined the four most common bridge types and developed a fragility curve for each of them. Zanini et al. [42] developed a fragility curve for the typical bridge at risk of corrosion in the northeastern Italian transportation network. In the second part of their study, they used the curves to determine the vulnerability of the whole network, taking into account the change in traffic flow when necessary. Stefanidou and Kappos [37] applied generic fragility curves to the larger landmark bridges and concluded that generic curves are applicable to simply supported bridges, while they tend to over- or underestimate the seismic resilience of more complex bridge structures. They provide the detailed state of the current state of practice in fragility curve development based on the selected element, limit state, analysis method, and seismic input [37].

While they do not use fragility curves, Sheikh and Légeron [43] have proposed a method for seismic assessment of bridges designed according to the Canadian Highway Bridge Design Code where the design method is force-based and strength is the measure of seismic performance. They developed a PGA-to-displacement ratio to replace the design code force-to-displacement ratio in order to take into account the seismic characteristic of the transportation network. In Italy, new “Guidelines for risk classification, safety assessment and structural health monitoring of existing structures” were recently adopted, including seismic assessment, and are presented on a Case Study PSC bridge by Cosenza and Losanno [44].

Di Sarno et al. [45] conducted a study on the seismic behavior of RC and Masonry bridges after the 2016 series of earthquakes in the Italian Apennine region. At that time, three large earthquakes with magnitudes M_L_ = 6.1, M_L_ = 5.9, and M_L_ = 6.5, each with several aftershocks, occurred over the period of August to October 2016. They concluded that the masonry bridges proved to be robust enough to withstand the seismic effects without global damage to the structure. The only bridges that were closed to traffic were those where the wing walls of the abutments were damaged, which was noted after the 2020 earthquakes in Croatia as well. Unreinforced masonry arch bridges are widely used in the European road and rail network and their assessment has been the subject of numerous studies, with several methods for their assessment developed over the last 25 years [31]. Due to their short spans and large overall mass and section dimensions, these bridges are generally very susceptible to the out-of-plane mechanism that can lead to complete bridge failure, as in the case of the Rio Claro Bridge in Chile [45]. Most commonly used analytical approaches to the assessment of masonry arch bridges are provided and compared in a study by Pelà, Aprile, and Benedetti [28]. Implementation of experimental testing for calibration of the numerical model of masonry arch bridges is presented in [26].

In addition to the displacement-based seismic capacity for existing RC piers, Huang and Huang [46] investigated other failure modes in their study of aging bridges in seismically active areas. They defined a practical framework for developing fragility curves for multiple failure modes (e.g., shear, bending shear, bending failure, etc.) based on the FE model, which was verified by six cyclic loading experiments. To simulate the deterioration mechanism (chloride infusion degradation), they used a probabilistic approach to reduce the cross-sectional area and yield strength of the corroded reinforcement. Alipour and Shafei [47] addressed the seismic resistance of deteriorated highway transportation networks. They used a nonlinear pushover analysis to estimate the decrease in the base shear capacity of the RC over a fifty-year lifespan due to corrosion of the reinforcement. The average decrease is presented in ten-year intervals, with the total decrease after fifty years being about 51%. Additional findings on the seismic resilience of concrete structures under corrosion can be found in [42,48]. Fragility curves are used to present the efficiency of the retrofitting strategy in a study by Padgett and DesRoches [41], in which they calculated the increase of the bridge’s resilience due to the selected retrofitting measures and limit state.

In the Croatian coastal region, there are a number of long-span RC arch bridges built between the 1960s and 2000s that are exposed to a very aggressive environment due to the strong winds and chlorides from the sea. As the older bridges (e.g., the Pag Bridge and the Šibenik Bridge) were constructed with only moderate seismic loads, there was a need for a practical and efficient seismic load assessment method. A multi-step method was developed in [27], with the first step being linear multimodal analysis, followed by the limit state checks. If the bridge does not meet the requirements, the evaluation is performed again at the second stage, where nonlinear pushover analysis is used. As the spandrel piers of certain RC arch bridges have non-typical cross-sections, they were assessed using both numerical and experimental approaches with scale models subjected to vertical and horizontal forces in the laboratory [6].

Zelaschi, Monteiro, and Pinho [49] used a different approach to assess the seismic fragility of transport networks at the macro level. They do not use the classical approach of a generic fragility curve, rather applying a statistical tool to develop a large-scale seismic assessment of RC bridges in Italy. Using the known geometric and material properties of the bridges in the network, they use parametric characterization and Latin hyper-cube sampling to define a two-variable formula for the preliminary estimation of the period of vibration and seismic stress for selected bridges. While this approach presents an interesting method for large-scale assessment, the results are biased based on the uncertainty modelling, as is often the case with probabilistic methods. 

## 3. Seismic Retrofitting of Existing Bridges

After successfully assessing and evaluating the existing bridge at risk of seismic activity, the optimal retrofit strategy must be selected, a process that differs significantly from the design process for a new bridge. Therefore, the selection of a strategy must be made with the aim of minimizing construction work and traffic disruption on the bridge, keeping the overall cost in reasonable proportion to the value of the bridge, and providing the retrofitted bridge with adequate seismic resilience [50]. The cost–benefit analysis of bridge assessment, maintenance, and repair is a topic that has been studied by a number of authors, and will not be further developed in this paper as it is beyond our scope. For more information on estimating the value of bridges as part of the transportation network, see, e.g., [51]; for the cost optimization algorithm for bridge repairs, see, e.g., [1].

After an extensive literature review of the topic of retrofitting methods was conducted, the majority of the papers focus either on a certain retrofitting method, e.g., jacketing [52], or a certain bridge type, e.g., arch bridges [50]. Therefore, state-of-the-art review presented here includes all available seismic retrofitting methods for road bridges. The list, along with corresponding literature sources, is summarized in Table 2 based on the structural element the method refers to. The most vulnerable structural elements of the bridge and corresponding retrofitting methods are described in detail in Section 3.1, Section 3.2 and Section 3.3, with practical examples where available.

### 3.1. Bridge Columns/Piers

The most common technique for seismically strengthening RC columns is to physically increase their cross-section and increase their ductility by adding encasement jackets made of new reinforced concrete, steel, or high-performance materials. Frequently used materials are Carbon Fiber Reinforced Polymer (CFRP), Aramid Fiber Reinforced Polymer (AFRP), Glass Fiber Reinforced Polymer (GFRP), Fiber Reinforced Cementitious Matrix (FCRM), Engineered Cementitious Composite (ECC), Ultra-High-Performance Fiber-Reinforced Concrete (UHPRC), etc.; a detailed review of jacketing techniques and the materials used can be found in [52].

**Figure 2:**
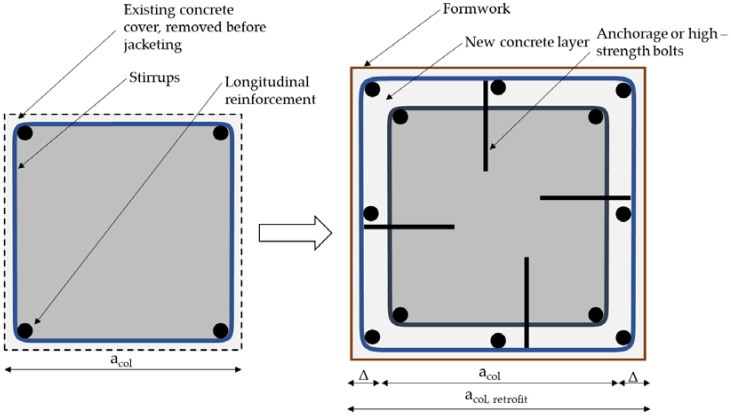
An example of reinforced concrete jacketing of existing columns.

The principle of this technique is quite simple; the objective is to increase the ductility and shear capacity of the existing column either by adding new reinforcement and a new layer of concrete or by using prefabricated jackets connected to the existing column by grouting. However, it is important to consider the increase in mass and stiffness of the retrofitted section, as their increase leads to shorter periods and increased base shear, thus increasing the seismic demands on the selected column and structural system. Traditionally, concrete jacketing is the oldest and most common method. A new layer of reinforcement is placed around the existing section over part or all of the length of the RC column and the concrete is poured using traditional formwork. The connection between the old and new sections is achieved by anchoring the new reinforcement in the existing concrete or by using high-strength bolts. An example of concrete encasement can be found in Figure 2.

**Figure 3:**
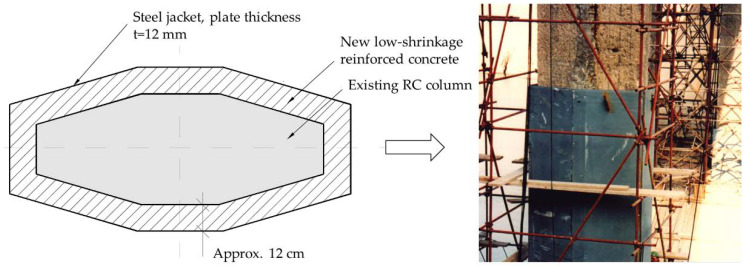
Seismic retrofitting of RC columns on Pag Bridge in Croatia showing the combination of RC and steel jacketing.

The obvious disadvantages of RC jacketing are its cost and time inefficiency due to formwork installation, especially for taller piers. Moreover, the increase in ductility depends only on the additional longitudinal and transverse reinforcement (stirrups), as concrete is a brittle material. Another disadvantage of this technique is the relatively large increase in cross-section compared to other methods, which can be crucial in existing bridges where the passage under the bridge for crossing roads, railways, etc. is restricted. Taking into account the typical cross-sections of bridge piers and the diameter of the reinforcement used, the thickness of the cross-section with the RC sheathing increases by about 0.5 m. On the other hand, steel or high-performance material sheathing can increase the ductility and resilience of the cross-section by only a few centimeters [54]. Steel jacketing is most effective on circular columns where the sheathing is prefabricated in two halves that are positioned and welded around the column. The gap between the steel plate and the existing concrete is filled with grout to ensure the bond. In this way, a new composite cross-section is created that features increased shear strength, ductility, and buckling resistance [65]. The disadvantage of steel jacketing is its lack of cost-effectiveness due to more complex procedures and corrosion protection. Moreover, similar to RC jacketing, it increases the stiffness of the cross-section, which changes the seismic demands on the pier/column [52]. There are some examples of the combined use of RC and steel jacketing, where the steel plates are used as formwork. This method was applied to the columns of the Pag Bridge in Croatia, as shown in Figure 3. The Pag Bridge, built in the late 1960s, is a concrete arch bridge with very slender columns in a very aggressive maritime environment. During major reconstruction in the late 1990s, these columns were strengthened by adding a new layer of reinforcement and concrete encased in a steel plate with a thickness of 12 mm [88].

In addition to traditional RC and steel jacketing, high-performance materials based on fiber-reinforced polymers (FRP) have become an increasingly popular method for retrofitting existing RC columns over the past three decades. The obvious advantages of these materials are speed and simplicity of installation, a high strength-to-weight ratio, and minimal increase in the cross-section of the structural element. Therefore, columns retrofitted with FRP materials do not have a significant increase in stiffness or mass, and the aesthetics of the bridge remain visually unchanged. Furthermore, these materials are environmentally friendly when compared to traditional reinforced concrete or steel. On the other hand, the efficiency of these materials is lower due to premature bonding; their utilization is only 30–35% [52]. Bonding is achieved externally using epoxy resins.

As shown in Table 2, there are a number of different FRP composites used for retrofitting RC columns, each with certain advantages and disadvantages. In addition to those listed, several composites are in various research phases and have not yet been used in practice. A comparison of different jacketing methods can be found in the detailed review by Raza et al. [52], which describes all common methods along with their effects on the structural element, cost, and other characteristics. Recapitulation of the three jacketing methods is provided in Table 3, based on [52].

### 3.2. Cap Beams/Concrete Joints

**Figure 4:**
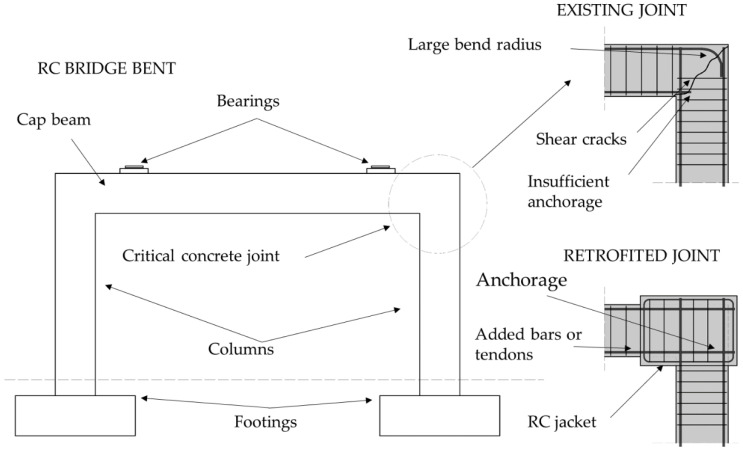
Seismic retrofitting of cap beams and concrete joints in RC bridge bents.

Many road bridges are supported on RC bents consisting of two or more columns connected with a transverse cap beam, as shown in Figure 4. The concrete joint between the top of the column and cap beam is subjected to both shear and flexural stresses during cyclic seismic loading. In older bridges, this joint is often designed without sufficient anchorage length in the longitudinal direction and sufficient reinforcement in the transverse direction, and can therefore be designated as a critical point because of the possibility of brittle failure. As described in Chapter 2, the approach to bridge design is that the superstructure should remain in the elastic region to force plastic hinges in the columns. This is achieved by increasing the stiffness and strength of the cap beam and the concrete joint between the beam and the column [65]. The retrofitting technique is similar to RC jacketing of columns, as the new concrete layer is poured around the joint along with sufficient reinforcement to be anchored in both the beam and a column; an example is shown in Figure 4.

RC bents for new bridges are not generally designed as shown in Figure 4 because the cap beam is usually extended beyond the columns, which provides more space for the reinforcement of the critical joint section. Therefore, while on some older bridges the cap beams are retrofitted similar to Figure 4, the new beam section is extended beyond the columns, creating a cantilever. This method is called a seat extender [53].

The lateral stiffness of the cap beam can be increased by external prestressing, a strength enhancement technique normally used for flexural strengthening of the superstructure [89]. An alternative approach to increasing the lateral stiffness of RC bents is to provide X bracing between the columns or to construct infill walls connected to both the columns and the cap beams. These retrofit methods are rarely used today because they are costly and affect both mass and stiffness; however, examples can be found among older bridges in the USA [53,65].

### 3.3. Seismic Isolation/Damping

Seismic isolation, often referred to as the most effective and successful structural earthquake protection measure, is now commonly used for all types of structures in seismically active areas. The principle is very simple, aiming to “decouple” the ground motion and vibration of the structure and thereby reduce the lateral forces on the structure. The concept of seismic isolation is several centuries old, and is not described in this paper; it can be found in a detailed report by Makris [72].

In the design and the assessment of road bridges, seismic isolator bearings (SIB) are placed between the superstructure and the substructure, and can be positioned under the foundations of the piers as well, though this is less common. The principle is to reduce the seismic response of the bridge by “decoupling” the superstructure and substructure, thus reducing the displacement of the superstructure and consequently the lateral forces in the piers and abutments. Both experimental and analytical studies have proven that there is a substantial decrease in the seismic response of the isolated and non-isolated bridge [76,79]. The disadvantage of isolation is that while it reduces the stiffness and corresponding lateral forces, it increases the natural period and total displacements of the structure. To account for this, all SIBs are equipped with built-in or external energy dissipators called dampers that reduce the Eigenperiod by about 30% [78].

The most common bridges where seismic isolation has been used for retrofitting are those with massively heavy superstructures supported on a relatively slender substructure (piers and abutments). Prior to the introduction of design codes with integrated seismic loads, most bridges were supported on unreinforced elastomeric bearings used primarily to transfer extensive vertical gravitational and traffic loads and horizontal loads due to thermal expansion and creep. Therefore, in the event of an earthquake there was a risk of brittle failure of piers due to extensive lateral forces, especially for irregular continuous bridges where the piers have a significant height difference. Application of seismic isolation in the USA was initiated after the 1971 San Fernando earthquake, and was widely accepted in the practice with new Caltrans guidelines in the aftermath of the Loma Prieta earthquake in 1989 [71].

**Figure 5:**
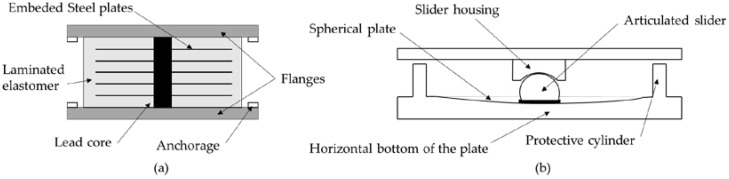
Isolation bearing schematics (**a**) elastomer (rubber)-based; (**b**) friction (sliding)-based.

SIB can be divided into two main groups, those based on elastomers (rubber-based) and those based on friction (sliding-based), depending on the principle of energy dissipation. The elastomer-based bearings are more commonly used for simply supported bridges, while plain bearings (also known as pot bearings) are used for longer continuous bridges spanning multiple spans. A schematic of each type is shown in Figure 5.

The selection of bearings for seismic isolation in bridge retrofitting depends on numerous bridge characteristics, such as the structural system, number, height of piers, etc. The dimensions of the bearings depend on the required vertical load-carrying capacity (bearing dimensions) and the allowable total displacement (bearing height). A number of authors have performed both numerical and experimental analyses of commonly used SIBs, e.g., [75,76,78,79,90]. Compared to the previously-listed retrofit techniques, seismic isolation is the most effective in terms of cost–benefit analysis, although replacement of the bearings requires complete closure of the bridge. In addition, many older bridges do not have columns designed for the installation of a hydraulic jack to elevate the superstructure during replacement. Therefore, the piers or cap beams on these bridges must be reinforced prior to seismic isolation.

An alternative measure to mitigate lateral forces on longer bridges is the installation of dampers, which are referred to as “passive energy dissipation devices”. However, dampers are mainly used in the construction of new landmark bridges or to control the vibration of railway bridges carrying high-speed trains. They are rarely used for retrofitting typical road bridges (see e.g., [13,81,82,83]). The first Case Study is an example of a seismic retrofit using dampers for restriction of longitudinal displacement of the superstructure.

Seismic restrainers and bumper blocks can be considered as a retrofit technique for RC bridges, where severe displacement of the superstructure can cause the girders to slip off the bearings. Bumper blocks are installed in most new bridges in seismic areas as a protective measure and to increase structural robustness [4]. Restraint systems are a protective measure that prevents the superstructure from slipping off its supports, and are one of the most cost-effective retrofit techniques. Cable restraints were used extensively on California bridges after the San Fernando earthquake [65]. Restrainers are suitable for bridges with multiple simply supported spans because they “couple” the spans together in both the longitudinal and transverse directions without affecting the structural system.

There are a number of other methods that are used today for existing bridges, more commonly for special bridges (masonry, cable-stayed bridges, listed bridges, etc.) which are beyond the scope of this paper. Several of them are listed in Table 2, together with relevant sources offering a more detailed review.

## 4. First Case Study Bridge

### 4.1. General Bridge Description

**Figure 6:**
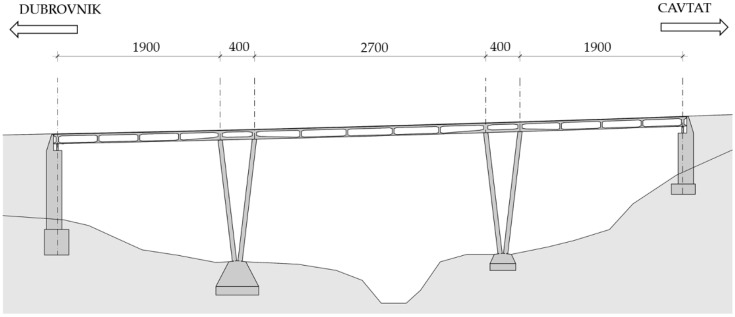
Longitudinal section of the first Case Study bridge (units in cm).

**Figure 7:**
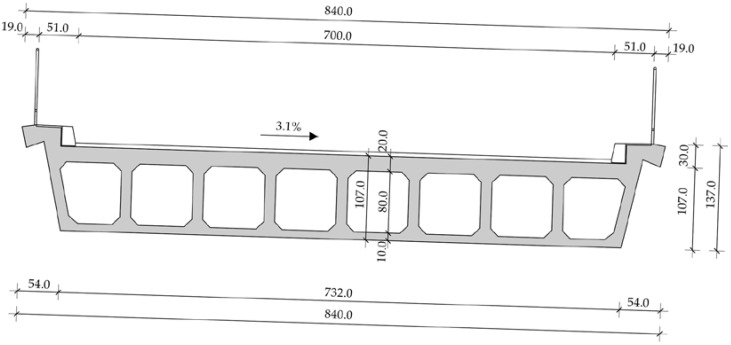
Cross-section (in the middle of the span) of the first Case Study bridge (units in cm).

The first Case Study [13] bridge is a reinforced concrete bridge constructed monolithically in the 1960s. The bridge is comprised of five spans with dimensions of 19.0 + 4.0 + 27.0 + 4.0 + 19.0 m, and follows a complex road axis that features horizontal and vertical curves. The superstructure is comprised of a voided RC slab supported on V-shaped piers, visible in Figure 6; a cross-section of the slab is presented in Figure 7.

The connection between the superstructure and the abutments is considered hinged, as the slab is supported on “Pendl” bearings. However, the connection of the slab and V-shaped piers (four in total) is hinged only in the longitudinal direction; in the transversal direction it is rigid and the two piers and deck form a rigid frame. The resulting statical system of the bridge is called a hinged strut frame.

The bridge was designed according to 1960s codes prior to the Skopje earthquake in 1963, after which the design codes were updated; thus, seismic loads were not taken into account. A review of the available documentation shows that only dead loads, traffic loads, temperature variations, and concrete shrinkage were considered.

### 4.2. Assessment Procedure

The height of the voided slab is 110 cm, the concrete class is C20/25, and the reinforcement is smooth with a yield strength of 220/360 N/mm^2^. In the middle of the span, the bottom flange has a thickness of 10 cm and is reinforced with three layers of Ø20 mm plain bars; above the supports, the flange is 20 cm thick and is reinforced with both Ø10 and Ø20 mm bars. The top flange has a constant thickness of 20 cm and is reinforced with both Ø12 and Ø16 mm bars in the middle of the span and Ø20 mm bars above the supports. The webs have a constant thickness of 14 cm and are reinforced with Ø12 bars.

The cross-section of V-shaped piers has a constant depth of 50 cm with variable width, ranging from 100 cm at the bottom to 200 cm at the top of the piers. The longitudinal reinforcement is comprised of a total of 30 Ø20 bars; the stirrups are positioned with variable spacing ranging from 7 to 30 cm. Additional stirrups are placed at the same spacing for the confinement of the longitudinal bars. All stirrups in both superstructure and piers have a diameter of 8 mm.

**Figure 8:**
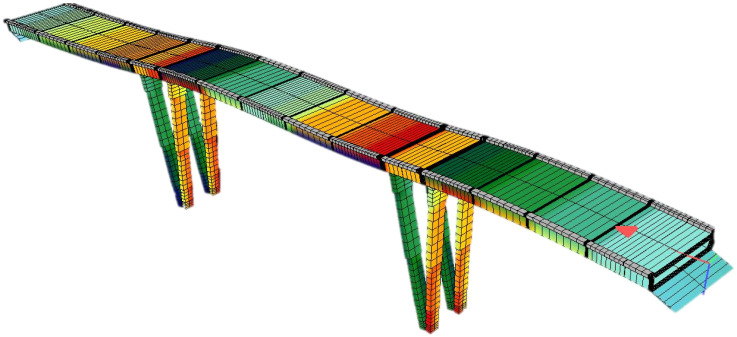
Numerical model of the Case Study bridge, first modal shape (longitudinal).

The bridge was assessed for both modern-day traffic loads and for seismic effects, as it is located in an earthquake-prone area in the southern coastal part of Croatia where the peak ground acceleration is 0.290 g for a return period of 475 years and the PGA is 0.146 g for a 95-year return period. As the traffic load assessment exceeds the scope of this paper, only seismic assessment and retrofitting proposals are presented. The numerical model of the bridge was developed using software for structural analysis and beam elements for both superstructure and piers. All bearings were modelled as springs with variable longitudinal and transverse stiffness to simulate the realistic behavior of the bridge. In accordance with EN 1998-3 [16], the cracked state of concrete was taken into account by a reduction in the structural stiffness. To simulate this reduction, the concrete modulus of elasticity in the numerical model was reduced to 50% of its value. The numerical model is presented in Figure 8.

As the connection between the piers and superstructure is hinged in the longitudinal direction, the longitudinal resulting force is transferred to abutments. The seismic analysis was conducted both using linear modal analysis and nonlinear static pushover analysis, using the multi-level method developed by Franteović et al. [27] in accordance with current design codes. In the first step of the analysis, the target displacement for both the longitudinal and transverse direction was obtained using linear modal analysis. A total of fifty modal shapes were calculated; the results show that the first dominant mode is shaped as a translation in the longitudinal direction, while the second (with a smaller period) is a translation in the transverse direction. The first modal shape is presented in Figure 8, while the resulting modal parameters (periods, target displacements, and response spectrum values for obtained periods) for the first two modal shapes are provided in Table 4.

**Figure 9:**
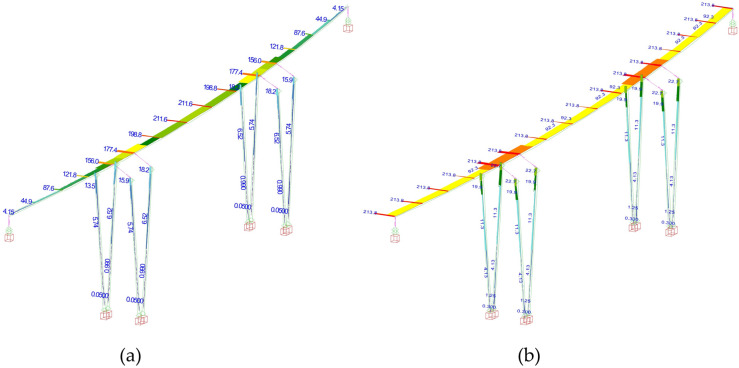
(**a**) horizontal load distribution along the superstructure; (**b**) horizontal load in proportion with dominant modal shape.

The target displacement for the first modal shape (Table 1) is 76 mm; while the existing expansion joint was designed to allow a maximum longitudinal displacement of 70 mm, when exceeded this would cause a collision between the superstructure and abutment wall. Therefore, the Case Study bridge was reassessed using the static nonlinear pushover analysis. A total of four cases were calculated with two types of load distributions for each direction, as presented in Figure 9 for first modal shape.

**Figure 10:**
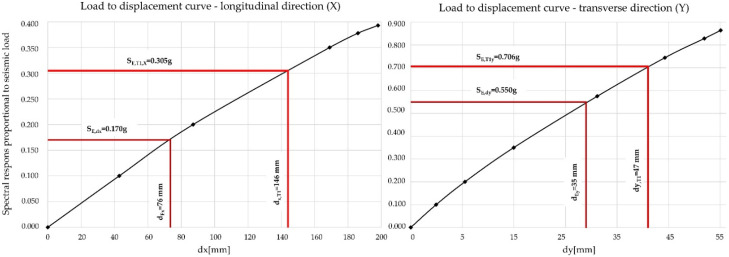
Load–displacement curves for the First Case Study bridge, longitudinal (**left**) and transverse (**right**) direction.

Pushover analysis results are presented in the form of load–displacement curves for each direction in Figure 10; the less favorable of two load distribution cases are presented. Two spectral responses are provided for each modal shape; the first (S_E,dx_ and S_E,dy_) shows the seismic load in which the target displacements (d_Ex_ and d_Ey_) are reached, while the second (S_E,T1,x_ and S_E,T1,y_) shows the maximum seismic load based on linear analysis along with the corresponding maximum displacements (d_x,T1_ and d_y,T1_).

The curves in Figure 10 show that the Case Study bridge does not have sufficient seismic resilience, as displacements corresponding to the two dominant modal shapes (Tx and Ty) exceed the target displacements (d_x,T1_ > d_Ex_ and d_y,T1_ > d_Ey_ ).

In addition to the displacements, the load-carrying capacity of the piers, abutments, and bearings was assessed. The pier shear failure occurs when the maximum seismic load is applied, while the “Pendl” bearings in the abutments cannot withhold the resulting forces.

### 4.3. Retrofitting Proposal

In accordance with the assessment results (Figure 10), a proposal for seismic retrofitting is presented [13], focusing on the limitation of the displacements and the shear failure of the piers.

**Figure 11:**
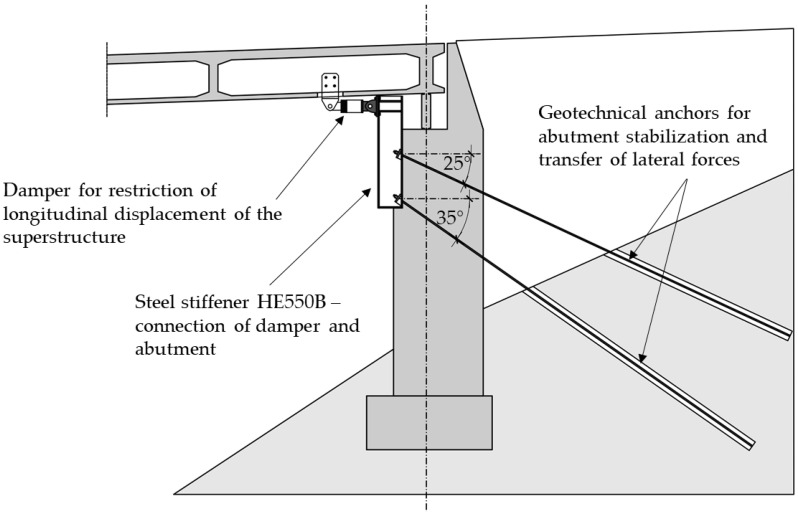
The proposed damper installation for the restriction of longitudinal displacement with geotechnical anchors, first Case Study bridge.

In order to restrict the longitudinal displacement of the superstructure, a total of three dampers with a maximum +/−50 mm should be placed on the abutment which transfers the longitudinal reaction, as shown in Figure 10. Each of the dampers has a capacity for transfer of longitudinal force of 1250 kN; however, analysis of the existing abutments showed that such loads exceed its load-bearing capacity. Therefore, the abutment should be strengthened; this can be conducted using RC jacketing with additional reinforcement or with the addition of geotechnical anchors which would transfer the load directly to the embankment. Due to the limited space beneath the bridge, the solution with geotechnical anchors was chosen, as can be seen in Figure 11.

**Figure 12:**
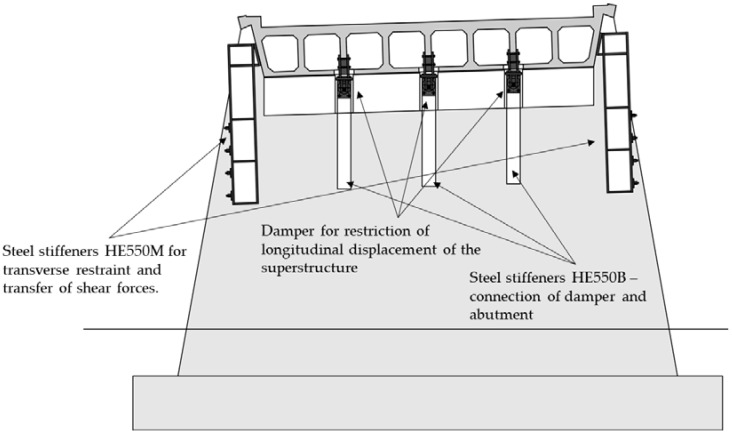
Transverse view of the abutment with both longitudinal and transverse stiffeners and displacement restraints, proposal for seismic retrofitting of the first Case Study bridge.

As the supports on the abutment are not able to withstand the transverse seismic forces, a transversal displacement restraint to be placed on both abutments is proposed. These are present in Figure 12 as additional steel profiles (HE550M) which have sufficient shear resistance to withstand imposed seismic forces. The shear strengthening of the piers should be conducted using FRP jacketing, as RC (due to limited space) and steel (due to variable cross-section) are not optimal solutions and would not be cost-effective.

## 5. Second Case Study Bridge

### 5.1. Rapid Visual Assessment after the December 2020 Earthquake

**Figure 13:**
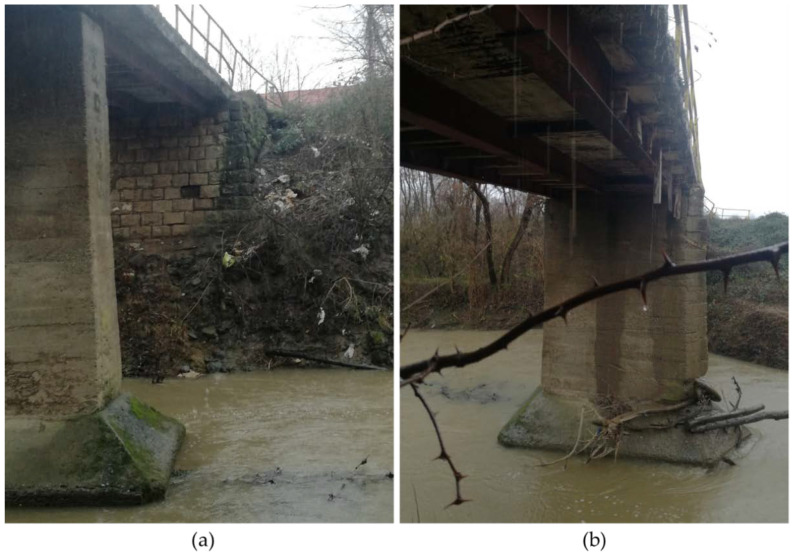
(**a**) Masonry abutment and (**b**) composite superstructure of the bridge to Majske Poljane.

The bridge over the Maja River at the southern entrance to the village of Majske Poljane was a three-span continuous girder bridge built in the 1950s, although this is based on conjecture as no data were available. The bridge superstructure consists of three cast-iron girders and a monolithic reinforced concrete deck resting directly on two RC piers and abutments. The abutment towards the town of Glina is masonry, the other is reinforced concrete. The superstructure and the abutment are presented in Figure 13.

Nine months after the Zagreb earthquake, Sisak Moslavina County was hit by two strong earthquakes, the first, in the early morning of 28 December, had a magnitude of M_L_ = 5.4, and the next day an even stronger earthquake struck, with a magnitude of M_L_ = 6.2 and an intensity between VIII and IX according to the EMS−98 scale. The resulting damage was devastating and claimed seven lives. The village of Majske Poljane suffered the most damage; almost all houses were destroyed. Immediately after the first earthquake, teams of structural engineers were sent out to conduct a preliminary rapid assessment of the structural damage using the procedures introduced after the Zagreb earthquake, as described in [91]. The authors were tasked with the preliminary rapid assessment of all bridges in the area [14], as heavy construction equipment, trucks with humanitarian aid, etc., were needed.

**Figure 14:**
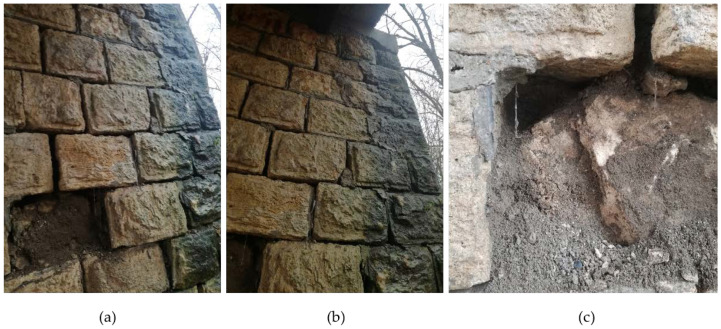
(**a**,**b**) Damage to the masonry walls of the abutment; (**c**) escarpment of infill material from the abutment.

During the visual inspection of the bridge to Majske Poljane, it was found that the masonry abutment had structural damage affecting its stability, with immediate action needed in order to allow heavy traffic over the bridge. The damage to the abutment is shown in Figure 14. The stone blocks from both the abutment wall and the wing walls were damaged and started to fall off. As the abutment was constructed as shallow with land infill that to transfer the vertical loads to the foundations, there was a danger that the collapse of the wing walls would result in the collapse of the bridge.

### 5.2. Urgent Retrofitting and Decision for Further Actions on the Bridge

**Figure 15:**
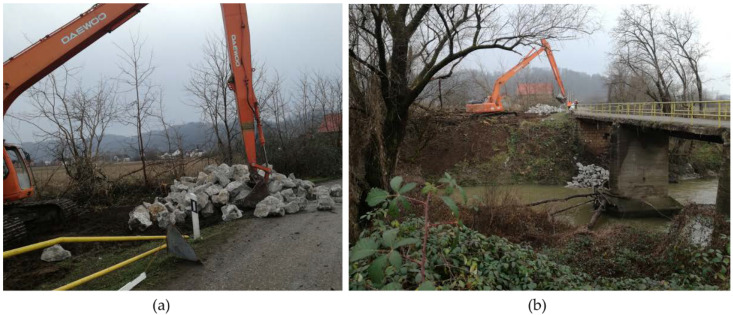
(**a**) Stone material for urgent stabilization; (**b**) placing of the additional weight on the abutment and embankment.

Both the wing and abutment walls required stabilization, and the only possible solution due to urgency and the available resources in the area at the time was to impose an additional dead load on the abutment and surrounding embankment. This was conducted by adding stone blocks that would act as stabilizing weights to allow traffic on the bridge, which was critical as the bridge is one of the only two entrances to the village. This urgent and temporary retrofitting is shown in Figure 15.

In addition to the damage caused by the earthquake, it was clear that the bridge was in an overall poor condition due to irregular maintenance and scour damage. Therefore, several months after the earthquake, when the surrounding area stabilized, a detailed inspection and assessment were conducted. As the bridge is currently under construction, the details of the project cannot be disclosed.

## 6. Conclusions

This paper reviews the state of the art in seismic assessment and retrofitting methods for existing road bridges, an important challenge for engineers which is not covered by the Eurocode.

There are a number of methods available for analysis of the structural behavior and response of road bridges during a seismic event; a list is provided in Table 1, with no general conclusion as to the optimal choice.

**Figure 16:**
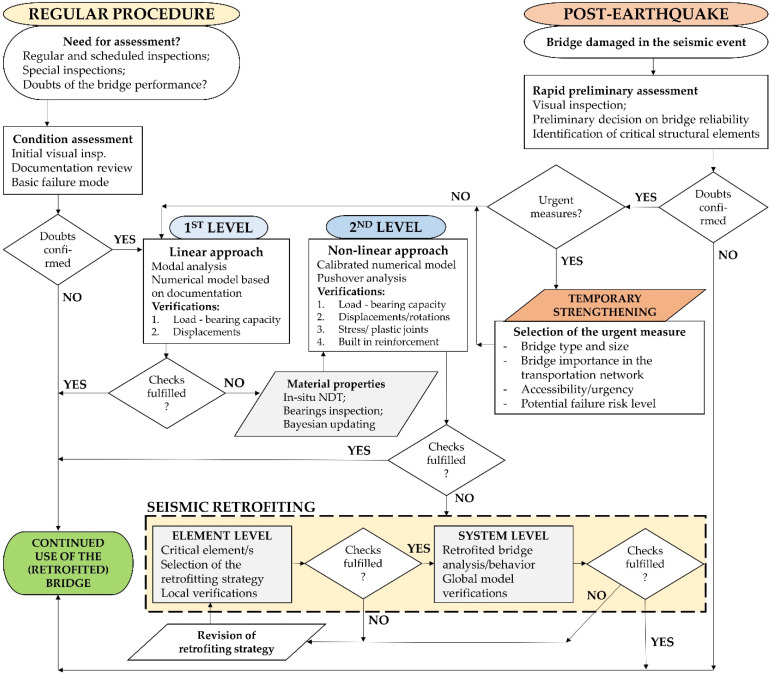
Proposed multi-level assessment method for typical existing road bridges.

The multistep procedure for the seismic assessment of typical existing road bridges is defined and presented, and is shown in the flowchart in Figure 16. The procedure is based on a step-by-step analysis in cases both where a bridge is inspected as part of regular maintenance (as in the first case study) and where inspection is urgent due to a recent severe seismic event (as in the second case study). In both instances, the first step in the analysis is a visual inspection, followed by a decision as to whether further evaluation is required. If it is, the bridge is assessed at the first level by developing a numerical model based on documentation and on-site measurements and using linear modal analysis. For bridges damaged by recent seismic activity, there is an additional step where decisions as to urgent stabilization measures precede the first assessment level. 

If a bridge meets the required ULS checks (codified-EN 1998-2 [5]), it is considered reliable and can be used without restrictions. However, if it does not meet the ULS checks, it is re-evaluated at the second level, where the numerical model is calibrated using material properties obtained with in situ NDT tools and Bayesian updating. The analysis at this level is based on a nonlinear pushover analysis, and additional checks are performed. Similar to the first level, the bridge is considered safe for use if it meets the verifications. If not, the seismic retrofit strategy is selected and applied based on the identification of the critical structural elements. First, the local checks (at the element level) are performed, and if they are satisfied, evaluation at the system level (global model) is performed. If at any of the steps the selected measure is not considered effective, the procedure is repeated with a revised retrofit strategy.

The retrofitting methods for existing road bridges in earthquake-prone areas are summarized in Table 2, based on the corresponding structural element. The most vulnerable parts of the common bridge types are piers and bearings, as they are sensitive to lateral loads caused by seismic events.

As a continuation of this research, fragility curves will be developed for the bridges in Sisak-Moslavina County to be used for priority ranking. However, the detailed assessment and retrofitting for those bridges which are deemed as vulnerable requires a more site-specific approach, similar to the second Case Study bridge.

The new generation of Eurocodes currently in development are expected to be expanded, and will have more focus on pre-existing structures. Existing bridges, both road and railway, should be covered in detail in terms of both analysis and retrofitting methods. It is recommended that modern materials based on polymers are included, along with ultimate and serviceability limit state verification, as this would expand their practical application to real bridges.

## Figures and Tables

**Table 2:** List of common methods for seismic retrofitting of existing road bridges.

Retrofit Method	Bridge Element	Bridge Type	Source
Steel jacketing	RC piers	Any bridge with RC piers	[52,53,54,55]
Concrete/mortar jacketing			
CFRP jacketing			[52,54,55,56,57]
ECC jacketing			[58,59]
AFRP jacketing			[52,60]
FRCM jacketing			[57,61]
GFRP jacketing			[52]
UHPFRC jacketing/repair			[62,63,64]
Bracing or infill walls between piers in the transverse direction			[65]
External prestressing with unbonded tendons	Superstructure	girder bridge, cable stayed bridge, slab bridge, box-girder bridge,	[13,66,67]
Span restrainers		any bridge with sliding bearings	[53,65]
Reinforced concrete jacking	Cap beams/ RC joints	Any bridge with RC piers/cap beams	[53,65,68,69,70]
Transverse external prestressing			[66,67]
Seat extenders	Cap beams/Abutments		[53,65]
Seismic isolation	Bearings	All bridges	[71,72,73,74,75,76,77,78,79]
Foundation cap confinement			[65]
Restrainers			[65,80]
Bumper blocks			[4,53]
Dampers			[13,75,81,82,83]
Replacement			[76,84,85,86]
Spandrel wall strengthening	Spandrel walls	Masonry bridges	[50,87]
Abutment wing walls stabilization	Abutment	Any bridge with massive abutments	[53]

**Table 3:** Comparison of the main characteristics of the described jacketing methods.

Method	Effect on the Structural Element			Cost
	Strength	Ductility	Stiffness	
RC jacketing	Increase	Increase	Increase	Very high
Steel jacketing	Significant increase	Significant increase	Increase	High
FRP jacketing	Increase	Significant increase	No effect	Moderate

**Table 4:** Modal parameters for first Case Study bridge, obtained using linear analysis.

Modal Shape	Direction	Period	Spectral Response	Target Displacement
1	Longitudinal (x)	T_x_ = 0.94 s	S(T_x_) = 0.305 g	d_E,x_ = 76 mm
2	Transverse (y)	T_y_ = 0.36 s	S(T_y_) = 0.706 g	d_E,y_ = 35 mm

## Data Availability

Not applicable.
